# Supplementary material for: Complexome profiling of the Chlamydomonas psb28 mutant reveals TEF5 as an early PSII assembly factor
Source: Plant Cell. 2025 Mar 17;37(6):koaf055. doi: 10.1093/plcell/koaf055 (PMC12164590; doi:10.1093/plcell/koaf055)
Supplement: koaf055_Supplementary_Data [file koaf055_supplementary_data.zip › psb28_interactive.zip!/plots/intensity/qualityPlots/peptides/MPPA1_Cre17.g722800.t1.2.html]

### Cre17.g722800.t1.2

Trivial name: MPPA1  
SeqCoverage [median]: 10.50  
Mass: 54.28 kDa  
  
 

| Sequence | MissedCleavage | Mass [Da] | wtIntensity | muIntensity | Score |
| --- | --- | --- | --- | --- | --- |
| ALVELAAPMLAGLPK | 0 | 1492.87 | 384.04 | 0.00 | 93.10 |
| SKPSLAAYGDNTQSLDPSVLAR | 1 | 2289.16 | 0.00 | 1621.95 | 70.76 |
| TYVEMLEAVTADDVRK | 1 | 1838.91 | 0.00 | 1642.40 | 44.93 |
|  |  |  | 384.0 | 3264.4 |  |
